# The CALERIE^™^ Genomic Data Resource

**DOI:** 10.1101/2024.05.17.594714

**Published:** 2024-08-22

**Authors:** CP Ryan, DL Corcoran, N Banskota, Indik C Eckstein, A Floratos, R Friedman, MS Kobor, VB Kraus, WE Kraus, JL MacIsaac, MC Orenduff, CF Pieper, JP White, L Ferrucci, S Horvath, KM Huffman, DW Belsky

**Affiliations:** 1. Robert N. Butler Columbia Aging Center, Columbia University Mailman School of Public Health, New York, NY, USA; 2. Department of Genetics, University of North Carolina at Chapel Hill, Chapel Hill, NC; 3. Intramural Research Program of the National Institute on Aging, NIH - Baltimore, MD-USA; 4. Department of Systems Biology, Columbia University Irving Medical Center; 5. Biomedical Informatics Shared Resource, Herbert Irving Comprehensive Cancer Center, Columbia University Irving Medical Center; 6. Department of Biomedical Informatics, Columbia University Irving Medical Center; 7. BC Children’s Hospital Research Institute, University of British Columbia, Vancouver, BC V5Z 4H4, Canada; 8. Department of Medical Genetics, Faculty of Medicine, University of British Columbia, Vancouver, BC V6T 2A1, Canada; 9. Centre for Molecular Medicine and Therapeutics, Vancouver, BC V5Z 4H4, Canada; 10. Child and Brain Development Program, Canadian Institute for Advanced Research, Toronto ON M5G 1M1, Canada; 11. Edwin S. H. Leong Centre for Healthy Aging, University of British Columbia, Vancouver, BC; 12. Duke Molecular Physiology Institute, Duke University School of Medicine, Durham, NC 27701, USA; 13. Department of Medicine, Duke University School of Medicine, Durham, NC 27701, USA; 14. Duke Center for the Study of Aging and Human Development, Duke University School of Medicine, Durham, NC 27701, USA; 15. Dept of Biostatistics and BioInformatics, Duke University School of Medicine, Durham, NC, USA.; 16. Human Genetics, David Geffen School of Medicine, UCLA, Los Angeles, USA; 17. Department of Epidemiology, Columbia University Mailman School of Public Health, New York, NY, USA

## Abstract

Caloric restriction (CR) slows biological aging and prolongs healthy lifespan in model organisms. Findings from CALERIE-2^™^ – the first ever randomized, controlled trial of long-term CR in healthy, non-obese humans – broadly supports a similar pattern of effects in humans. To expand our understanding of the molecular pathways and biological processes underpinning CR effects in humans, we generated a series of genomic datasets from stored biospecimens collected from n=218 participants during the trial. These data constitute the first publicly-accessible genomic data resource for a randomized controlled trial of an intervention targeting the biology of aging. Datasets include whole-genome SNP genotypes, and three-timepoint-longitudinal DNA methylation, mRNA, and small RNA datasets generated from blood, skeletal muscle, and adipose tissue samples (total sample n=2327). The CALERIE Genomic Data Resource described in this article is available from the Aging Research Biobank. This mult-itissue, multi-omic, longitudinal data resource has great potential to advance translational geroscience.

## Introduction

Caloric restriction (CR) involves a reduction of caloric intake by 10–20% or more while maintaining adequate protein and micronutrient levels^1^. In laboratory experiments, CR slows biological aging and prolongs lifespan in multiple model organisms^2^. In humans, observational studies of individuals who self-impose CR^3,4^, short-term trials involving participants with obesity^5^, and involuntary CR among participants in the Biophere-II experiment^6^ broadly support some of the health and longevity promoting effects shown in model organisms^7^. The CALERIE-2^™^ (Comprehensive Assessment of Long-term Effects of Reducing Intake of Energy) study (NCT00427193 at clinicaltrials.gov), is the first-ever randomized controlled trial of CR in healthy, non-obese humans. CALERIE-2^™^ is a phase II, multicenter, randomized controlled trial (RCT) that tested the effects of a 24-month CR intervention in healthy non-obese adult men and women. Although the trial did not achieve durable modification of its primary endpoints, resting metabolic rate and core temperature^8^, analysis of participants’ clinical laboratory values broadly support the hypothesis that CR promotes healthy aging; participants randomized to the CR condition experienced broad improvements in their cardiometabolic health relative to those in the control “ad libitum” (AL) condition^9^ and exhibited a slowed pace of biological aging, as measured from algorithms based on clinical laboratory values^10,11^. These observations motivated efforts to elucidate underlying cellular-level mechanisms, including through projects funded by the National Institute on Aging at Columbia Univeristy, Duke University, and the University of California Los Angeles. The outcomes of these projects, including SNP genotype, DNA methylation, and messenger and small RNA sequencing data derived from blood, muscle, and adipose tissues collected from participants at pre-intervention baseline and at the 12- and 24-month follow-up assessments comprise the the CALERIE^™^ Genomic Data Resource.

### Description of the Trial

Extending CALERIE^™^ phase I^12^ in both scale and duration, CALERIE-2^™^ recruited a total of 220 subjects and assigned them in a 2:1 allocation to a CR treatment group or ad libitum (AL) control arm^13^. Subjects were randomly-assigned to CR or AL groups stratified on study site, sex, and body mass index. Participants in the CR group were assigned to a protocol designed to result in a 25% reduction in caloric intake relative to estimated energy requirements at enrollment. CR participants received an intensive behavioral intervention that included individual and group sessions, a meal provision phase, digital assistants to monitor caloric intake, and training in portion estimation and other nutrition and behavioral topics^14^. Adherence was assessed using measures of energy expenditure using the doubly-labelled water method as well as expected changes in body composition. The duration of the study for both CR and AL participants was 2-years.

**Figure 1.**
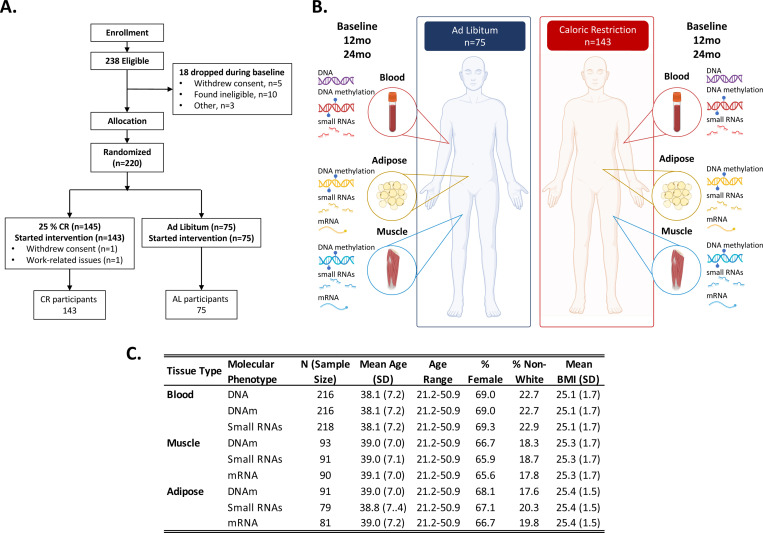
Study design, participant information, and overview of molecular datasets generated for the CALERIE^™^ caloric restriction trial. Panel A shows the Consort diagram showing enrollment, eligibility, and final sample sizes by treatment group. Panel B shows a schematic of molecular data types and tissue sources. Genetic material (DNA) was derived from blood samples for both ad libitum (AL) and caloric restriction (CR) groups at baseline. DNA methylation was derived from blood, muscle, and adipose samples for both AL and CR at baseline, 12-months, and 24-months. Small RNAs were derived from blood, muscle, and adipose samples for both AL and CR at baseline, 12-months, and 24-months. mRNA was derived from muscle and adipose samples for both AL and CR at baseline, 12-months, and 24-months. Panel C shows a table of demographic information for participants that contributed data to individual tissue level molecular datasets. Total sample size, mean and standard deviation in age, age range, percent female, percent who self-reported as other than white, and mean and standard deviation of BMI. Figure created using images from Biorender.com

Throughout the 2-year study duration, starting at baseline prior to randomization and recurring at months 1, 3, 6, 9, 12, 18, and 24, participants were evaluated for a range of pre-specified anthropometric, psychological, and physiological outcomes^8^. Blood samples were collected every six months. At baseline, 12-months, and 24-months, whole blood and samples were collected and banked. In addition, a subset of participants agreed to biopsies of adipose and muscle tissue at baseline, 12-months, and 24-months. From these samples SNP-based genotypes, DNA methylation, mRNA, and small RNAs were assayed. Here, we describe these datasets and provide an overview of this data resource (Fig. 1).

More details about the CALERIE^™^ trial, including study protocols and on-going and published research, are available at https://calerie.duke.edu. Data can be accessed through the Aging Research Biobank (https://agingresearchbiobank.nia.nih.gov/studies/calerie/). Data use is restricted to non-commercial use in studies to determine factors that affect age-related conditions. Biospecimens are available, limited to research on effects that caloric restriction may have on aging and aging-related diseases.

## Results

### Overview

**Figure 2.**
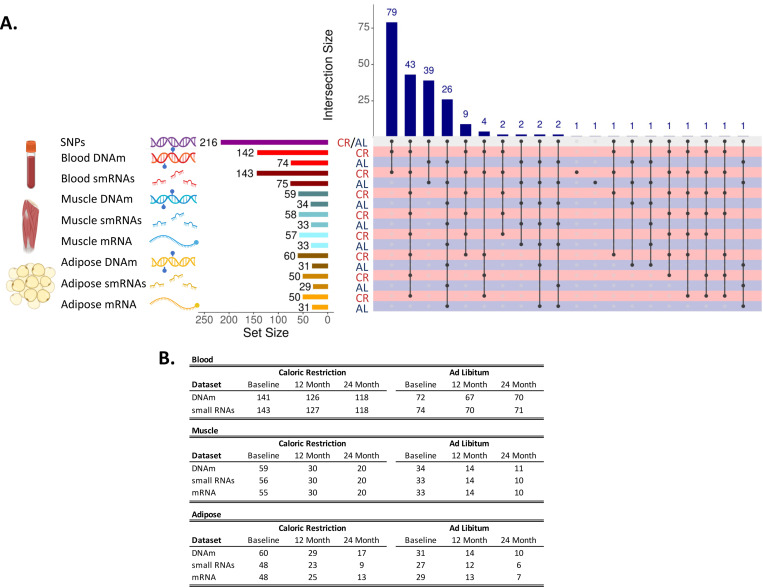
Panel A shows an upset plot showing overlap of available datasets across treatment group, tissues, molecular data type. The left-hand side shows tissue type (blood, muscle, or adipose), type of molecular data type (SNPs, DNAm, smRNAs, or mRNA), set size for each tissue and data type combination in number of unique individuals, and group (CR or AL). The bottom right-hand side shows points and connecting lines indicating overlapping intersections across tissues and data types color coded by treatment group (CR = red, AL = navy). The top right hand side shows a barchart indicating sample sizes for the overlapping intersections of tissue and datatypes. Each tissue and molecular data type combination is linked to a corresponding color scheme as follows: genomic variation (purple DNA), blood DNA methylation (red DNA with lollipops), blood small RNAs (smRNAs; dark red RNA fragments), muscle DNA methylation (blue DNA with lollipops), muscle smRNAs (blue RNA fragments), muscle mRNA (blue single RNA strand), adipose DNA methylation (yellow DNA with lollipops), adipose smRNAs (yellow RNA fragments), adipose mRNA (yellow single RNA strand). Panel B shows Individual sample numbers by molecular data type, treatment group (Caloric Restriction or Ad Libitum) and follow-up visit (Baseline, 12 Month, or 24 Month) across different tissue types. For some individuals, samples were available at follow-up but not baseline (or vice versa), so baseline numbers for a tissue and treatment group combination will not always match sample sizes in Figure 2 Panel A. Figure created using images from Biorender.com.

Of 238 eligible enrolled participants, n=220 were allocated to the trial and randomized to CR and AL treatment groups (Fig. 1). Detailed study procedures were published previously^8,12,15^ and are described here in brief. Two participants in the CR treatment group withdrew from the study, leaving n=143 participants in the CR group and n=75 in the AL group. In total, genomic data was produced for n=218 unique individuals. SNP data were available for 216 participants. DNAm and RNA sample coverage varied by treatment group, time-point, and tissue type (Fig. 2 and Supplemental Figure S1). A sample availability matrix for all samples by molecular data type, tissue, and timepoint is available in Table S1.

### Genotyping

**Figure 3.**
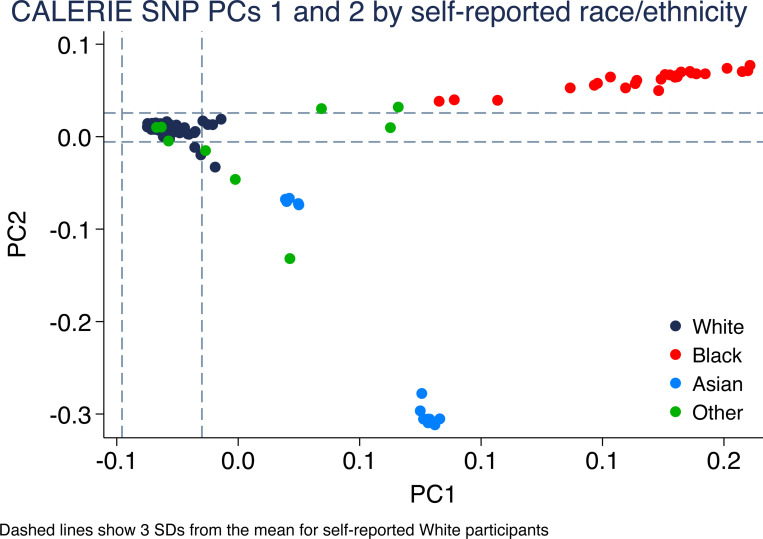
SNP-based principal components scores for CALERIE^™^ participants. The figure shows the top two SNP-based principal components (PCs) for CALERIE^™^ participants (n=217), colored by self-reported race/ethnicity. Dashed lines indicate three standard deviations from the mean for participants who self-reported race/ethnicity as White. The top-5 SNP-based principal-components explained 11.9% (PC1) 4.8% (PC2), 1.5% (PC3), 1.4% (PC4), and 1.3% (PC5) of the genetic variation among CALERIE participants.

Genotypic data is available for all but two participants who were randomized, provided consent, and completed the trial (n=216). Figure 3 Panel A illustrates genetic ancestry of CALERIE participants as reflected in the first two principal components of the genome-wide single-nucleotide polymorphism (SNP) data. Figure 3 Panel B illustrates the percent genetic variation explained by the top 10 genetic principal components.

### DNA methylation

DNA methylation (DNAm) was assayed from DNA extracted from whole blood, skeletal muscle, and adipose tissue using Illumina EPICv1 arrays (Fig. 1B). Assays of whole blood DNAm were completed by the Kobor Lab at the University of British Columbia as part of NIH grant R01AG061378. Assays of muscle and adipose DNAm were completed by the UCLA Neuroscience Genomics Core (UNGC) as part of NIH grant U01AG060908 using DNA extracted from banked tissue by the VB Kraus lab in the Duke Molecular Physiology Institute. Quality control and preprocessing for muscle and adipose tissue DNAm was carried out by the Geroscience Computational Core of the Robert N. Butler Columbia Aging Center.

Blood DNAm is available for at least one timepoint for n=216 participants (n=142 participants in the CR group and n=74 in the AL group)^16^. Sample sizes for blood DNAm for individual timepoints by treatment group are provided in Fig. 2A. Muscle DNAm is available at least one timepoint for n=93 (n=59 participants in the CR group and n=34 in the AL group). Sample sizes for muscle DNAm for individual timepoints by treatment group are provided in Fig. 2A. Adipose DNAm is available for at least one timepoint for n=91 participants (n=60 participants in the CR group and n=31 in the AL group). Sample sizes for adipose DNAm for individual timepoints by treatment group are provided in Fig. 2A. Muscle and adipose DNAm data have not been published previously.

To facilitate the rapid appraisal of the underlying data structure and missingness, we generated a series of summary datasets. For each tissue, we provide a set of tables listing the probes that passed quality control (**Supplemental Data Files 1–4, 9–12, 17–20**) and their beta-value distributions and missingness at each timepoint (**Supplemental Data Files 5–8, 13–16, 21–24**).

In addition to CpG methylation levels, we also computed a series of composite variables from the blood DNAm data. To help account for technical variability across samples, we provide the top principal components of EPIC-array control-probe beta values for blood, adipose, and muscle^17^. For contrast between tissues, we illustrate deconvoluted cell types (adipose, epithelial, fibroblast, and immune cells) for blood, muscle, and adipose using hierarchical in Figure S2.

**Figure 4.**
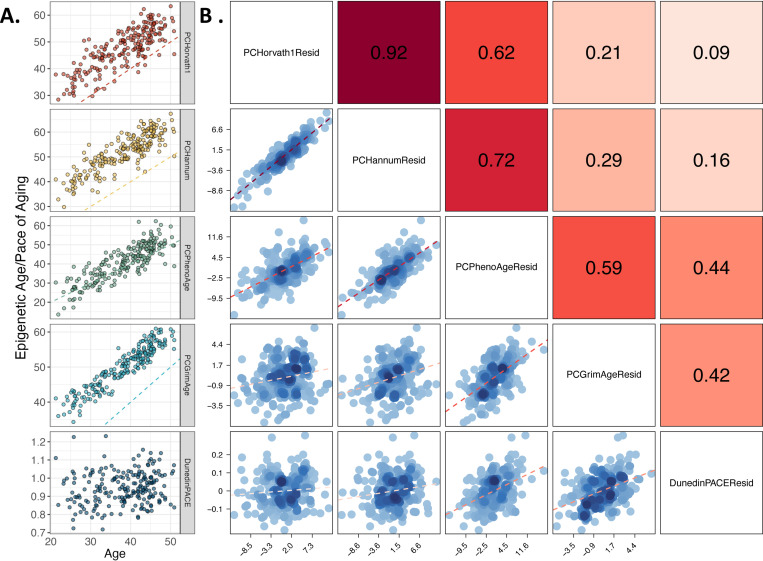
Associations of DNA methylation measures of aging with chronological age and age-residualized DNA methylation measures of aging with each other. Panel A shows DNA methylation measures of aging (Y-axis) against chronological age (X-axis) for n=212 men and women at pre-intervention baseline. The dashed colored line on each facet is the line of identity (intercept=0, slope=1), indicating where predicted epigenetic age or pace of aging would equal chronological age. Correlations with chronological age are as follows: PC Horvath Clock r=0.84, PC Hannum Clock r=0.88, PC PhenoAge Clock r=0.85, PC GrimAge Clock r=0.92, DunedinPACE r=0.15. Panel B shows correlations between age-residualized DNA methylation measures of aging for n=212 men and women at pre-intervention baseline with each other. The dashed red line on each facet is the fitted regression slopes. Pearson correlations between DNA methylation measures of aging are shown on the upper diagonal facets, with the shade of the facet indicating the strength of the correlation.

**Figure 5.**
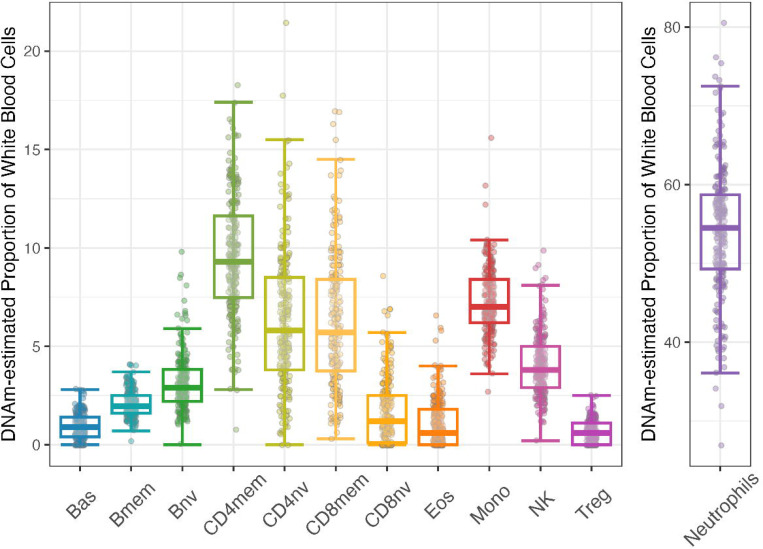
Relative proportions of 12 white blood cell types estimated for all participants at baseline. The figure shows the estimated relative proportions of 12 white blood cell types for all participants at baseline, prior to treatment (Bas=basophils, Bmem=memory B cells, Bnv=naïve B cells, CD4mem=memory CD4T cells, CD4nv=naïve CD4T cells, CD8mem=memory CD8T cells, CD8nv=naïve CD8T cells, Eos=Eosinophils, Mono=Monocytes, NK=Natural Killer cells, Treg=T regulatory cells, Neutrophils=Neutrophil cells). Individual points represent individual observations. Box and whisker plots showing median (horizontal line), 25^th^ quartile (bottom of the box), 75^th^ quartile (top of the box), and minimum and maximum values within 1.5 × IQR (interquartile range; top and bottom segments, respectively). Neutrophils are plotted on a separate scale.

We also include in this database the series of epigenetic clocks and estimated white blood cell proportions reported previously^18^ as well as a newly-derived set of estimated white blood cell proportions^19^. Intercorrelations of chronological age and five epigenetic clocks (PCHorvath, PCHannum, PCPhenoAge, PCGrimAge, and DunedinPACE) at the baseline assessment, prior to treatment, are provided in Figure 4. Intercorrelations of chronological age and ten epigenetic clocks (Horvatth, PCHorvath, Hannum, PCHannum, PhenoAge, PCPhenoAge, GrimAge, PCGrimAge, and DunedinPACE) at the baseline assessment, prior to treatment, are provided in Figures S3 and S4. As published elsewhere, the clocks show low to moderate intercorrelation with one another, reflecting the different methods of their construction^20,21^. Relative proportions of each of the 12 white blood cell types estimated at baseline measurement, prior to treatment, using previously described methods^19^ are graphed in Figure 5.

### RNAseq

RNA sequencing was performed for three tissues: plasma, skeletal muscle, and adipose tissue (Fig. 1B). All RNA was extracted from plasma at the Duke Molecular Physiology Institute from stored plasma, skeletal muscle, and adipose-tissue biospecimens. Plasma and adipose RNA were sequenced at the Duke University Center for Genomic and Computational Biology. Muscle RNA was sequenced at the Center for Cancer Research Sequencing facility (National Cancer Institute). Messenger RNA (mRNA) and small RNA (smRNA) datasets were generated for skeletal muscle and adipose tissues. Only smRNAs were generated from plasma due to mRNA sample quality issues.

#### Small RNAs.

Plasma smRNAs were available for at least one timepoint for n=218 participants (n=143 in the CR group and n=75 in the AL group). Sample sizes for plasma smRNAs for individual timepoints by treatment group are provided in Fig. 2A. Muscle smRNAs are available for at least one timepoint for n=91 participants (n=58 in the CR group and n=33 participants in the AL group). Sample sizes for muscle smRNA for individual timepoints by treatment group are provided in Fig. 2A. Adipose smRNAs are available for at least one timepoint for n=79 participants (n=50 in the CR group and n=29 participants in the AL group). Sample sizes for adipose smRNAs for individual timepoints by treatment group are provided in Fig. 2A.

#### Messenger RNA.

**Figure 6.**
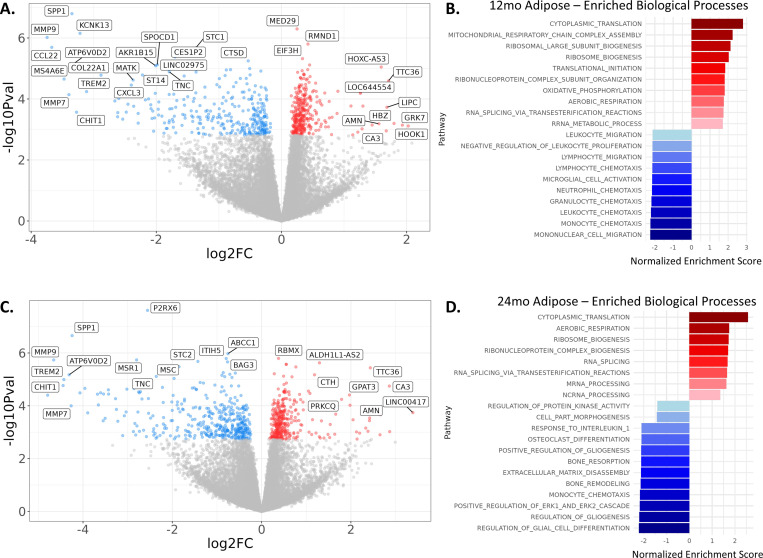
Differences in adipose mRNA expression (A and C) and enriched biological processes (B and D) between CR and AL groups at the 12-month and 24-month timepoints. For Panels A and C, x-axis shows log2 fold-change differences between groups. The y-axis shows -log10 p-value. Blue points show genes with false-discovery rate q-values less than 0.05 were downregulated in CR participants. Red points show genes with false-discovery rate q-values less than 0.05 were upregulated in CR participants. Gray points show genes with q-values equal or greater than 0.05. Using a false-discovery cutoff of q=0.05, a total of 605 genes (from 19471 genes total) were differentially-expressed between CR and AL treatment groups at the 12-month timepoint (n=34, Panel A) and 734 genes were differentially-expressed between CR and AL treatment groups at the 24-month timepoint (n=18, Panel C). A dispersed but otherwise arbitrary selection of 31 differentially-expressed genes labeled by gene symbol are shown for the 12-month treatment effect. A dispersed but otherwise arbitrary selection of 23 differentially-expressed genes labeled by gene symbol are shown for the 24-month treatment effect. Panels B and D show the top-10 upregulated (red) and down-regulated (blue) biological pathways from Gene Ontology based on overlapping results from both fast GSEA and GAGE gene set enrichment. The full set of enriched pathways for 12-month and 24-month follow-ups are in Tables S1 and S2, respectively.

Muscle mRNA is available for at least one timepoint for n=90 participants (n=57 in the CR group and n=33 in the AL group)^22^. Sample sizes for muscle mRNA for individual timepoints by treatment group are provided in Fig. 2A. Adipose mRNA is available at least one timepoint for n=81 participants (n=50 in the CR group and n=31 in the AL group). This dataset was generated from an independent and larger set of samples than what has been published previously^23^. Sample sizes for this new adipose mRNA for individual timepoints by treatment group are provided in Fig. 2A. Adipose mRNA analysis has not been published previously. An overview of adipose mRNA response to CALERIE intervention in this dataset is reported in Fig. 6. Differential expression analysis identified 605 genes modified by CR at the 12-month follow-up (Fig. 6A; 309 upregulated and 296 downregulated) and 734 genes at the 24-month follow-up (Fig. 6C; 330 upregulated and 404 downregulated) at the FDR corrected q-value of 0.05.

For the 12-month follow-up, pathway analysis of differentially-expressed genes identified 241 enriched biological pathways (12 upregulated, 229 downregulated). Upregulated pathways included those involved in mitochondrial function, enhanced protein synthesis through ribosomal biogenesis, and RNA processing. These pathways are consistent with improved cellular energy metabolism, reduction of oxidative stress, and improved cellular repair and gene regulation, supporting longevity promoting tissue health and function through improved cellular maintenance and repair. Downregulated pathways included those involved in immune system activation and inflammatory responses, cellular and ion homeostasis, and endocytosis and cellular response to lipids. These pathways are consistent with decreases in pro-inflammatory responses, and improved handling of ions and lipids, resulting in a reduction in stress-response pathways and inflammation thought to be central to the aging process. Moreover, these findings are consistent with a previous adipose RNAseq analysis from a subset of CALERIE participants^23^. Spadaro et al reported a similar induction of genes related to mitochondrial function and a suppression of genes related to inflammation^23^. Together, our current data, in corroboration with what has been previously reported, support the idea that caloric restriction appears to slow several hallmarks of aging in adipose tissue, including the loss of mitochondrial function and chronic inflammation^24^. A complete gene set enrichment for the 12-month follow-up is provided in Table S4. The top-10 upregulated and top-10 downregulated pathways for 12-month follow-up are illustrated in Figure 6B.

For the 24-month follow-up, pathway analysis of differentially-expressed genes identified 155 enriched biological pathway (8 upregulated, 147 downregulated). Upregulated pathways included aerobic respiration, enhanced ribosome production and protein synthesis, and gene expression regulation. These pathways are consistent with sustained improvements in cellular metabolism, repair, and gene regulation detected in adipose mRNA during the 12-month follow-up. Downregulated pathways at the 24-month follow-up also included many of the same pathways identified at the 12-month follow-up; those involved in immune system activation and inflammatory responses, cellular and ion homeostasis, and cellular metabolism. Additional pathways at the 24-month follow-up were involved in development and morphogenesis pathways, suggesting an additional benefit of CR on tissue composition and differentiation in the form of tissue maintenance. A complete gene set enrichment for the 24-month follow-up is provided in Table S5. The top-10 upregulated and top-10 downregulated pathways for 24-month follow-up are illustrated in Figure 6D.

To facilitate the rapid appraisal of the underlying data structure of smRNA and mRNA for plasma, adipose, and muscle samples, we generated a series of summary datasets for transcripts passing quality control and their distributions across each timepoint (**Supplemental Data Files 25–44**).

## Discussion

CALERIE-2^™^ is the first ever Randomized Control Trial (RCT) of long-term CR in healthy, non-obese humans. The CALERIE^™^ Genomic Data Resource reported here represents the first such resource for a trail of an intervention hypothesized to modulate biological processes of aging. Below, we briefly outline applications of this resource to advance the frontiers of translational geroscience.

First, the CALERIE^™^ Genomic Data Resource provides opportunities to compare impacts of novel drug and behavioral interventions designed to modulate aging biology with a gold-standard intervention well-established in the animal literature. Admittedly, CALERIE^™^ is an imperfect implementation of CR compared to the controlled laboratory settings of model organisms. Nevertheless, CALERIE^™^ provides a baseline against which alternative, potentially more scalable geroprotective interventions can be compared. The molecular functions, biological processes, and cellular components generated from genomic datasets provide a ‘common language’ for comparisons with other intervention and observational studies in humans^25^. For example, the CALERIE^™^ Genomic Data Resource will facilitate comparison with a growing list of behavioral and pharmacological intervention trials now underway, many of which have specified genomic variables as endpoints^26^. For example, blood DNA methylation (DNAm) are being generated in a range of intervention trials, including trials of diet and exercise^27–30^, supplements, including Vitamin D^31^ and nicotinamide mononucleotide^32^, and pharmacological interventions, including Dasatinib, Quercetin, and Fisetin^33^. RNA from blood, as well as RNA and DNAm from adipose and muscle are also available for a range of diet and exercise-related interventions^34–39^. As genomic data will become available for an increasing range of trials, the value of the CALERIE^™^ Genomic Data Resource will continue to grow.

Second, the CALERIE^™^ Genomic Data Resource will allow researchers to compare the molecular effects of CR on human subjects with the molecular responses of in vitro and in vivo model systems^40–42^. This complementary approach allows researchers to validate findings from rigorous human trails like CALERIE^™^ in laboratory settings and vice versa, and can provide insights into phylogenetically-conserved molecular responses to CR that can be used as benchmarks for interventions on biological aging. In one example, researchers examined the effect of CR in mice on muscle satellite cell expansion through the binding of circulating plasminogen to the Plg-RKT receptor. The role of plasminogen on muscle satellite cell expansion in mice was validated in humans using measures of blood plasminogen, a DNAm surrogate marker of plasminogen inhibitor PAI-1^43^, and muscle biopsies from the CALERIE^™^ trail^44^. Through the recapitulation of findings in mice and humans, the researchers were able to demonstrate the mediating role of the plasminogen system on muscle stem cell regeneration in response to CR. The parallel analysis of human and in vivo or in vitro models has been successfully employed in the study of cancer and other diseases^45,46^ and is likely to play a key role in the future of translational geroscience.

Third, the CALERIE^™^ Genomic Data Resource provides opportunities to evaluate the sensitivity of proposed omics-based indices of biological aging to CR intervention. There are now abundant data to establish that intervention in the CALERIE^™^ trial improved a range of health parameters for the treatment group^9–11,47,48^. These data provide a background against which to evaluate proposed novel indices of biological aging, as we did in our prior analysis of epigenetic clocks^18^. The CALERIE^™^ Genomic Data Resource will provide a platform for analysis of the many new blood-based clocks recently published and others still forthcoming^49–52^ as well as nonomics-based measures of biological aging^18,53,54^. Furthermore, DNAm and RNA from muscle and adipose data from the CALERIE^™^ Genomic Data Resource will provide researchers with the rare opportunity to study the relationship between new and existing blood-based measures and molecular responses to CR in other tissues.

Fourth, the CALERIE^™^ Genomic Data Resource will provide developers with a powerful setting in which to develop, implement, and validate new and existing multi-omics tools^55–57^. Multi-omics approaches leverage complementary information provided by different molecular data types to gain a more comprehensive understanding of complex biological systems and processes. Multi-omics tools use similarities, network structures, correlations, and probabilistic relationships across molecular datasets to generate novel insights not evident from analysis of individual omics data^58^. Most multi-omics tools have been developed and implemented in cancer research, where they are used for studying disease biology, disease subtyping, and in the development of diagnostic and prognostic biomarkers^58^. The application of such multi-omics tools in the CALERIE^™^ Genomic Data Resource could be used to better understand the complex biology of CR in living humans, the heterogeneity of responses to CR, or pharmacological targets for CR mimicking drugs.

Finally, the CALERIE^™^ Genomic Data Resource can provide researchers with preliminary data for planning interventions on biological aging. For example, CALERIE^™^ results for current epigenetic clocks^18^ highlight the need for large sample sizes in trials planning to use these measures as outcomes. CALERIE^™^ data can similarly illuminate sample size planning around other genomic measures of aging.

In conclusion, the CALERIE^™^ Genomic Data Resource is a new platform to elucidate the biology of caloric restriction and its effects on biological processes of aging. This resource can also contribute to the discovery and validation of aging biomarkers and provide a reference resource for new geroscience clinical trials. It is our hope that publication of omics datasets for geroscience trials will become standard practice, advancing open science within geroscience research and enabling rapid comparative and integrative analyses to accelerate translation of therapies to extend healthspan.

## Methods

### Study Protocol

CALERIE Phase 2 was a multi-center, randomized controlled trial conducted at three clinical centers in the United States^8^ (ClinicalTrials.gov Identifier: NCT00427193). It aimed to evaluate the time-course effects of 25% CR (that is, intake 25% below the individual’s baseline level) over a 2-yr period in healthy adults (men aged 21–50 yr, premenopausal women aged 21–47 yr) with BMI in the normal weight or slightly overweight range (BMI 22.0–27.9 kg m^−2^). The study protocol was approved by Institutional Review Boards at three clinical centers (Washington University School of Medicine, St Louis, MO, USA; Pennington Biomedical Research Center, Baton Rouge, LA, USA; Tufts University, Boston, MA, USA) and the coordinating center at Duke University (Durham, NC, USA). All study participants provided written, informed consent. Nongenomic data were obtained from the CALERIE Biorepository (https://calerie.duke.edu/apply-samples-and-data-analysis).

### Sample Collection

#### Blood Collection

Approximate 70ml of blood was collected from participants after an overnight fast (12 hours) and immediately cryopreserved in liquid nitrogen and stored at −80°C from the time of acquisition until further processing.

#### Muscle biopsies

Skeletal muscle biopsies of the vastus lateralis (VL) muscle were obtained using a 25-gauge, 2 inch needle as described in Bergstrom^59^. Participants were fasting overnight (12 hours) at the time of biosample collection. Briefly, the biopsy site was treated with local anesthesia. A small incision was made using a scalpel and the biopsy needle was inserted. Approximately 50–100 mg of muscle tissue was extracted and placed in a cryovial and immediately flash frozen in liquid nitrogen. Samples were stored at −80°C until processing.

#### Adipose biopsies

Abdominal subcutaneous adipose tissue biopsies were obtained using 3 or 4mm x 15cm liposuction needles. Participants were fasting overnight (12 hours) at the time of biosample collection. Briefly, an area approximately one hand-width lateral to the umbilicus was sterilized and local anesthesia obtained using 2% lidocaine. After making a small insicion with a scapel, the liposuction needle was inserted and suction was applied to the attached syringe to withdraw approximately 900mg of tissue. Tissue was rinsed with phosphate buffered saline, weighed, placed in a cryovial, and immediately flash frozen in liquid nitrogen. Samples were stored at −80°C until processing.

### Genotyping

The CALERIE^™^ genotype dataset was produced by the Kobor Lab at the University of British-Columbia and the Genomics Analysis Shared Resource at Duke University. DNA was extracted from n=217 baseline blood samples obtained from the CALERIE^™^ Biorepository at the University of Vermont. Participants were fasting overnight (12 hours) at the time of biosample collection. Genotyping was conducted using the Illumina Global Screening Array-24 v3.0 (GSA) BeadChips containing 654,027 markers, with ~30,000 add-on markers from Infinium PsychArray-24 focused content panel (Illumina, San Diego, CA). Briefly, 200ng DNA was processed and hybridized to the GSA chips according to the manufacturer’s instructions, and scanned using the Illumina iScan platform. Genotypes were called for SNPs matched to dbSNP v151 ^60^ and for which valid calls were made in >98% of participants using GenomeStudio v2.0 (Illumina, San Diego, CA). Additional SNPs were imputed using the IMPUTE2 software suite G3^61^ and the 1000 Genomes Phase 3 reference panel^62^. Pre-phasing and imputation were conducted by breaking the genome into 5 megabase fragments. The final SNP database consisted of directly genotyped SNPs and SNPs imputed with a >90% probability of a specific genotype for which call rates were >95% and minor allele frequencies were >1%.

#### SNP-principal components

The top 20 principal components were calculated on the autosomal SNPs using PLINK v1.90b3.36 with default parameters^63^.

### DNA methylation

#### Preprocessing, Normalization, and Quality Control

##### Blood

DNA methylation profiling for blood was conducted in the Kobor Lab (https://cmmt.ubc.ca/kobor-lab/) from whole-blood DNA stored at −80 degrees Fahrenheit. Briefly, 750ng of DNA was extracted from whole blood and bisulfite converted using the EZ DNA Methylation kit (Zymo Research, Irvine, CA, USA). Quantity and quality of the bisulfite-converted DNA using NanoDrop spectrophotometry. Methylation was measured from 160ng of bisulfite-converted DNA using the Illumina EPIC Beadchip (Illumina Inc, San Diego, CA, USA).

QC and normalization were performed using methylumi^64^(v2.32.0) and the Bioconductor (v2.46.0)^65^ package from the R statistical programming environment (v 3.6.3). Probes with detection p-values >0.05 were coded as missing; probes missing in >5% of samples were removed from the dataset (final probe n=828,613 CpGs). Normalization to eliminate systematic dye bias in the 2-channel probes was carried out using the methylumi default method.

##### Muscle

DNA methylation profiling for muscle was conducted in the UCLA UCLA Neuroscience Genomics Core (UNGC) from skeletal muscle DNA extracted in the VB Kraus lab at Duke University and stored at −80 degrees Celcius. Briefly, 250 ng of DNA was extracted from muscle tissue and bisulfite converted using the EZ DNA Methylation kit (Zymo Research, Irvine, CA, USA). Methylation was measured from 250 ng of bisulfite-converted DNA using the Illumina EPIC Beadchip (Illumina Inc, San Diego, CA, USA). Quality control and preprocessing for muscle tissue DNAm was carried out from IDATs in the Geroscience Core in the Butler Columbia Aging Center, and performed in the R computing environment using the same methods described for blood DNA methylation. The final probe number for muscle DNA methylation was n=861779.

##### Adipose

DNA methylation profiling for adipose was conducted in the UCLA Neuroscience Genomics Core (UNGC) from adipose tissue DNA extracted in the VB Kraus lab at Duke University and stored at −80 degrees Celcius. Briefly, 250 ng of DNA was extracted from adipose tissue and bisulfite converted using the EZ DNA Methylation kit (Zymo Research, Irvine, CA, USA). Methylation was measured from 250 ng of bisulfite-converted DNA using the Illumina EPIC Beadchip (Illumina Inc, San Diego, CA, USA). Quality control and preprocessing for adipose tissue DNAm was carried out from IDATs in the Geroscience Core in the Butler Columbia Aging Center, and performed in the R computing environment using the same methods described for blood and muscle DNA methylation. The final probe number for adipose DNA methylation was n=861714.

#### Derived Variables

##### DNAm control-probe principal components (PCs)

For blood, muscle, and adipose, we conducted principal component analysis of EPIC-array control-probe beta values to compute controls for technical variability across the samples. To do so, we loaded raw idats, normalized using *normalizeMethyLumiSet,* and extracted normalization quality control probes. Principal components (PCs) were extracted from normalization probes, and standard deviations of these PCs were standardized by squaring and dividing by the sum of their squares. PCs that explained 90% of the variance in normalization probes were retained.

##### Immune cell proportion estimates

For blood, proportions of CD8T, CD4T, natural killer cells, b cells, monoctyes, and neutrophils were estimated using the Houseman equation via the FlowSorted.Blood.EPIC package and standard “Blood” reference panel^66,67^(**Supplementary Data File 39**). An ‘extended’ version of *estimateCellCounts2* using *FlowSorted.BloodExtended.EPIC* using the “BloodExtended” reference panel was also used to calculate basophils, B naïve, B memory, CD4T naïve, CD4T memory, CD8T naïve, CD8T memory, eosinophils, monocytes, neutrophils, T regulatory cells and natural killer cells (**Supplementary Data File 40**). Cell estimates were estimated on a red green channel set (RGset) and noob normalized with the reference dataset as specified by the package developers^19^. For contrast between tissues, we illustrate deconvoluted cell types (adipose, epithelial, fibroblast, and immune cells) for blood, muscle, and adipose tissues using hierarchical EpiDISH^68^ in Figure S2.

##### Epigenetic clock estimates

Blood epigenetic clocks including Horvath, Hannum, PhenoAge, GrimAge were calculated using the matrix of beta values provided in the data repository and the online calculator (https://dnamage.genetics.ucla.edu/, since been superseded by https://projects.clockfoundation.org). DunedinPACE was calculated using the matrix of beta values provided in the data repository and the DunedinPACE R package (https://github.com/danbelsky/DunedinPACE). Principal components-based clocks (PC clocks) developed by Higgins-Chen and colleagues were calculated in R on noob normalized samples that passed sample quality control using previously described code and methods^69^.

### RNAseq

#### Blood

##### RNA extraction

Circulating RNA was extracted from 200 μL plasma using the Qiagen miRNeasy Serum/Plasma Advanced Kit (Catalog no. 217204). RNA samples were stored at −80°C until miRNA sequencing was performed.

##### sRNA sequencing

sRNA sequencing was performed on samples isolated from plasma (n=584; AL=36%:CR=64%). Sequencing libraries were prepared from 5μLs of miRNeasy RNA using the QiaSeq miRNA Library Auto Kit (384; cat # 331509) according to the manufacturer’s instructions. Library amplification (22 cycles) was performed on a Beckman i7 liquid handler, using QiaSeq miRNA 96 Index IL Auto A (384; cat # 331569). Library quality was evaluated using a fragment analyzer (Agilent); the library concentrations were quantified by Qubit ds DNA HS assay (ThermoFisher). Libraries were sequenced on a NovaSeq 6000 (Illumina) using S4 lanes and 75 base pair single reads.

##### sRNA processing and normalization

QIAseq smRNA sequencing FASTQ files were preprocessed using the GeneGlobe^®^ Data Analysis platform, Legacy version 2.0. SmRNA reads were first processed by trimming at the 3’ adapter using cutadapt. Next, the trimmed reads were used to identify insert and unique molecular index (UMI) sequences. Reads with <16 base pair insert sequences or less than 10 base pair UMI sequences were discarded. Annotations of insert and UMI sequences were mapped to the human reference genome (GRCh38/hg38) using the alignment tool, bowtie2 (v2.5.1). All miRNA/piRNA mapped reads and associated UMIs were then aggregated to count unique molecules using miRbase V21 and piRNABank, respectively. Following the data processing steps, UMI counts were filtered to remove low expression smRNAs (row sum counts <50 were removed). Filtered UMI counts were then used to calculate normalization factors using the trimmed mean of M-values (TMM) method within the BioConductor edgeR (v4.2.1) package.

#### Muscle

##### RNA extraction

Flash frozen skeletal muscle samples (~50 mg) were homogenized using a bead-based Qiagen TissueLyser II in l mL TRIzol^™^ Reagent for a total of 4 minutes, rotating the TissueLyser Adapter after 2 minutes (Invitrogen^™^ ThermoFisher #15596026). Following homogenization, RNA was extracted using a Qiagen RNeasy Mini Kit (Qiagen #74104) and stored at −80°C until mRNA sequencing.

##### mRNA sequencing

Illumina libraries were generated with the TruSeq Stranded Total RNA Library Preparation Kit. RNA was sequenced using the *Illumina NovaSeq 6000* sequencing system with paired-end reads. The samples yielded 387 to 618 million pass filter reads with more than 86% of bases above the quality score of Q30.

##### mRNA filtering, alignment, and genome annotation

The quality of Oreads in fastq RNA-Seq files were initially assessed using *FastQC* tool (v0.11.5; https://www.bioinformatics.babraham.ac.uk/projects/fastqc/), Preseq (v2.0.3)^70^, Picard tools (v2.17.11; https://broadinstitute.github.io/picard/) and RSeQC (v2.6.4)^71^. Reads were trimmed using bbduk (from bbtools package (https://jgi.doe.gov/data-and-tools/software-tools/bbtools/)^72^. Following trimming, cleaned reads were examined one more time using *FastQC.* Next, cleaned fastq files, along with reference human genome 38 and Ensembl annotation v104, were used as input for STAR (v2.7.10a), a splice-aware aligner implemented with a novel algorithm for aligning high-throughput long and short RNA-Seq data to a reference genome^73^. The STAR aligner was run with the –*quantmode TranscriptomeSam* parameter to also generate transcriptome BAM files, and was used for RSEM analysis. Genome BAM files were sorted and indexed using samtools. Finally, genome BAM files were used as input for featureCounts from the Rsubread package (v2.0.1)^74^, a suitable program for counting reads for various genomic features such as genes.

##### sRNA sequencing

sRNA sequencing was performed on samples isolated from muscle (n=116; AL=38%:CR=62%) using the same protocols as for blood, described above.

##### sRNA Processing and Normalization

sRNA data were processed and normalized using the same protocols as for blood, described above.

#### Adipose

##### RNA extraction

Segments of flash frozen abdominal adipose tissue (~50 mg each) were homogenized using a bead-based Qiagen TissueLyser II in l mL TRIzol^™^ Reagent for a total of 4 minutes, rotating the TissueLyser Adapter after 2 minutes (Invitrogen^™^ ThermoFisher #15596026). Following homogenization, RNA was extracted using the Qiagen RNeasy Mini Kit (Qiagen #74104) and stored at −80°C until smRNA sequencing.

##### mRNA sequencing

mRNA sequencing was performed on the Illumina NovaSeq 6000 Sequencer at the Duke Sequencing Core. Barcoded libraries for mRNA sequencing were produced from the Universal Plus mRNA-seq NuQuant kit (0408-A01, Tecan Genomics) according to manufacturers recommended protocol with 17 amplication cycles and 200ng of total starting RNA. The protocol was automated on a Perkin Elmer Sciclone G3 liquid handler.

##### mRNA filtering, alignment, and genome annotation

Alignment of fastq files against the GRCh38 assembly of the human genome was performed using Subread^75^ (v2.0.3) and bowtie2 (v2.5.1) on the Columbia Department of Systems Biology high performance cluster. Most samples aligned with >90% mapped reads. One sample aligned with 69.6% mapped reads, but demonstrated satisfactory base sequence quality, tile sequence quality, and per sequence quality scores. Quantification was performed using featureCounts^74^ method as implemented in Subread. A matrix of counts was constructed, with genes labeled using Entrez Gene ID numbers. Initial quality control steps and outlier detection was carried out in 5 groups of 30 samples each. Samples that passed the first screen were then combined for a final quality and outlier detection round. Quality was assessed and outliers detected by multidimensional scaling^76^ and hierarchical clustering^77^. Multidimensional scaling was performed using limma^78^ (v3.54.1). Hierarchical clustering was performed with Euclidean distance and complete linkage clustering^77^ as implemented in the Heatmap.2 command in gplots^79^. Optimal clusters determined by the gapstat^80^ method as implemented in NBClust^81^ (v3.0.1). During quality control, 2 samples – both baseline samples – were removed. One sample had too few reads, while the other was a clear outlier on multidimensional scaling plots and linkage clustering maps.

##### mRNA Differential Expression

Samples were normalized using the trimmed mean method^82^ and analyzed for two-sided differential expression with limma-voom with sample weighting to downweight the effect of sample outliers^78,83,84^. Participant was modeled as a random effect using the duplicate correlation method^85,86^. CR at follow-up (12- or 24-months) was compared to AL at follow-up (12- or 24-months), relative to CR at baseline compared to AL at baseline. Models included covariates for sex, age at baseline, BMI and RNA integrity number. Raw p-values were corrected for multiple testing using the Benjamini-Hochberg false discovery rate^87^. Limma estimates and effect-sizes are provided for 12-month and 24-month follow-up in Table S2 and Table S3, respectively.

For gene set enrichment analysis, we used a custom R function that integrates outputs from the fast GSEA (FGSEA; v1.30.0) and GAGE (v2.54.0) methodologies^88,89^. We focused on GO Biological Processes (GO-BP), with c5.go.bp.v7.5.symbols.gmt (downloaded from the Molecular Signature Database, http://www.gsea-msigdb.org/gsea/msigdb/) as our pre-defined gene set. FGSEA was executed with parameters set to a minimum pathway size of 15, a maximum of 600, and 10,000 permutations. Pathways with an FDR adjusted p-value below 0.05 were retained. To enhance the robustness of our findings, GAGE analysis was also performed, filtering pathways significant in both FGSEA and GAGE. We categorized pathways as either up-regulated or down-regulated based on their normalized enrichment scores and visualized the top 10 up- and down-regulated pathways.

##### sRNA sequencing

sRNA sequencing was performed on samples isolated from muscle adipose (n=65 AL=38%:CR=62%) using the same protocols as for blood, described above.

##### sRNA processing and normalization

sRNA data were processed and normalized using the same protocols as for blood, described above.

## Supplementary Material

Supplement 1

Supplement 2

Supplement 3

Supplement 4

Supplement 5

Supplement 6

## Data Availability

Processed data can be accessed through the Aging Research Biobank (https://agingresearchbiobank.nia.nih.gov/studies/calerie/). Data use is restricted to non-commercial use in studies to determine factors that affect age-related conditions. Original raw data may be obtained from the Belsky Lab (cac_geroscience@cumc.columbia.edu). Code used in the production of summary data and figures are available at https://github.com/CPRyan/CALERIE_Genomic_Data_Resource.

## References

[1] Bales C. W. & Kraus W. E. Caloric Restriction: IMPLICATIONS FOR HUMAN CARDIOMETABOLIC HEALTH. Journal of Cardiopulmonary Rehabilitation and Prevention 33, 201 (2013). 23748374 10.1097/HCR.0b013e318295019e PMC3696577

[2] Speakman J. R. & Mitchell S. E. Caloric restriction. Molecular Aspects of Medicine 32, 159–221 (2011). 21840335 10.1016/j.mam.2011.07.001

[3] Fontana L., Meyer T. E., Klein S. & Holloszy J. O. Long-term calorie restriction is highly effective in reducing the risk for atherosclerosis in humans. Proceedings of the National Academy of Sciences 101, 6659–6663 (2004). 10.1073/pnas.0308291101 PMC404101 15096581

[4] Meyer T. E. Long-Term Caloric Restriction Ameliorates the Decline in Diastolic Function in Humans. Journal of the American College of Cardiology 47, 398–402 (2006). 16412867 10.1016/j.jacc.2005.08.069

[5] Heilbronn L. K. Effect of 6-Month Calorie Restriction on Biomarkers of Longevity, Metabolic Adaptation, and Oxidative Stress in Overweight IndividualsA Randomized Controlled Trial. JAMA 295, 1539–1548 (2006). 16595757 10.1001/jama.295.13.1539 PMC2692623

[6] Walford R. L., Harris S. B. & Gunion M. W. The calorically restricted low-fat nutrient-dense diet in Biosphere 2 significantly lowers blood glucose, total leukocyte count, cholesterol, and blood pressure in humans. Proceedings of the National Academy of Sciences 89, 11533–11537 (1992). 10.1073/pnas.89.23.11533 PMC50586 1454844

[7] Flanagan E. W., Most J., Mey J. T. & Redman L. M. Calorie Restriction and Aging in Humans. Annu. Rev. Nutr. 40, 105–133 (2020). 32559388 10.1146/annurev-nutr-122319-034601 PMC9042193

[8] Ravussin E. A 2-Year Randomized Controlled Trial of Human Caloric Restriction: Feasibility and Effects on Predictors of Health Span and Longevity. The Journals of Gerontology: Series A 70, 1097–1104 (2015). 10.1093/gerona/glv057 PMC4841173 26187233

[9] Kraus W. E. 2 years of calorie restriction and cardiometabolic risk (CALERIE): exploratory outcomes of a multicentre, phase 2, randomised controlled trial. The Lancet Diabetes & Endocrinology 0, (2019). 10.1016/S2213-8587(19)30151-2 PMC6707879 31303390

[10] Belsky D. W., Huffman K. M., Pieper C. F., Shalev I. & Kraus W. E. Change in the Rate of Biological Aging in Response to Caloric Restriction: CALERIE Biobank Analysis. The Journals of Gerontology: Series A 73, 4–10 (2018). 10.1093/gerona/glx096 PMC5861848 28531269

[11] Kwon D. & Belsky D. W. A toolkit for quantification of biological age from blood chemistry and organ function test data: BioAge. Geroscience 43, 2795–2808 (2021). 34725754 10.1007/s11357-021-00480-5 PMC8602613

[12] Racette S. B. One Year of Caloric Restriction in Humans: Feasibility and Effects on Body Composition and Abdominal Adipose Tissue. The Journals of Gerontology: Series A 61, 943–950 (2006). 10.1093/gerona/61.9.943 PMC4376245 16960025

[13] Rochon J. Design and Conduct of the CALERIE Study: Comprehensive Assessment of the Long-term Effects of Reducing Intake of Energy. The Journals of Gerontology: Series A 66A, 97–108 (2011). 10.1093/gerona/glq168 PMC3032519 20923909

[14] Rickman A. D. The CALERIE Study: Design and methods of an innovative 25% caloric restriction intervention. Contemporary Clinical Trials 32, 874–881 (2011). 21767664 10.1016/j.cct.2011.07.002 PMC3185196

[15] Kraus W. E. 2 years of calorie restriction and cardiometabolic risk (CALERIE): exploratory outcomes of a multicentre, phase 2, randomised controlled trial. The Lancet Diabetes & Endocrinology 7, 673–683 (2019). 31303390 10.1016/S2213-8587(19)30151-2 PMC6707879

[16] Ramaker M. E. Epigenome-wide association study analysis of calorie restriction in humans, CALERIE TM Trial analysis. The Journals of Gerontology: Series A glac168 (2022) doi:10.1093/gerona/glac168. PMC9799188 35965483

[17] Lehne B. A coherent approach for analysis of the Illumina HumanMethylation450 BeadChip improves data quality and performance in epigenome-wide association studies. Genome Biology 16, 37 (2015). 25853392 10.1186/s13059-015-0600-x PMC4365767

[18] Waziry R. Effect of long-term caloric restriction on DNA methylation measures of biological aging in healthy adults from the CALERIE trial. Nat Aging 1–10 (2023) doi:10.1038/s43587-022-00357-y. 37118425 PMC10148951

[19] Salas L. A. Enhanced cell deconvolution of peripheral blood using DNA methylation for high-resolution immune profiling. Nat Commun 13, 761 (2022). 35140201 10.1038/s41467-021-27864-7 PMC8828780

[20] Belsky D. W. DunedinPACE, a DNA methylation biomarker of the pace of aging. eLife 11, e73420 (2022). 35029144 10.7554/eLife.73420 PMC8853656

[21] Belsky D. W. & Baccarelli A. A. To promote healthy aging, focus on the environment. Nat Aging 3, 1334–1344 (2023). 37946045 10.1038/s43587-023-00518-7 PMC12459207

[22] Das J. K. Calorie restriction modulates the transcription of genes related to stress response and longevity in human muscle: The CALERIE study. Aging Cell 22, e13963 (2023). 37823711 10.1111/acel.13963 PMC10726900

[23] Spadaro O. Caloric restriction in humans reveals immunometabolic regulators of health span. Science 375, 671–677 (2022). 35143297 10.1126/science.abg7292 PMC10061495

[24] López-Otín C., Blasco M. A., Partridge L., Serrano M. & Kroemer G. The Hallmarks of Aging. Cell 153, 1194–1217 (2013). 23746838 10.1016/j.cell.2013.05.039 PMC3836174

[25] Subramanian A. Gene set enrichment analysis: A knowledge-based approach for interpreting genome-wide expression profiles. Proceedings of the National Academy of Sciences 102, 15545–15550 (2005). 10.1073/pnas.0506580102 PMC1239896 16199517

[26] Moqri M. Biomarkers of aging for the identification and evaluation of longevity interventions. Cell 186, 3758–3775 (2023). 37657418 10.1016/j.cell.2023.08.003 PMC11088934

[27] Fiorito G. DNA methylation-based biomarkers of aging were slowed down in a two-year diet and physical activity intervention trial: the DAMA study. Aging Cell 20, e13439 (2021). 34535961 10.1111/acel.13439 PMC8520727

[28] Fitzgerald K. N., Campbell T., Makarem S. & Hodges R. Potential reversal of biological age in women following an 8-week methylation-supportive diet and lifestyle program: a case series. Aging 15, 1833–1839 (2023). 36947707 10.18632/aging.204602 PMC10085584

[29] Fitzgerald K. N. Potential reversal of epigenetic age using a diet and lifestyle intervention: a pilot randomized clinical trial. Aging 13, 9419–9432 (2021). 33844651 10.18632/aging.202913 PMC8064200

[30] McEwen L. M. DNA methylation signatures in peripheral blood mononuclear cells from a lifestyle intervention for women at midlife: a pilot randomized controlled trial. Appl. Physiol. Nutr. Metab. 43, 233–239 (2018). 29049890 10.1139/apnm-2017-0436

[31] Chen L. Effects of Vitamin D3 Supplementation on Epigenetic Aging in Overweight and Obese African Americans With Suboptimal Vitamin D Status: A Randomized Clinical Trial. The Journals of Gerontology: Series A 74, 91–98 (2019). 10.1093/gerona/gly223 PMC6612014 30256915

[32] Yi L. The efficacy and safety of β-nicotinamide mononucleotide (NMN) supplementation in healthy middle-aged adults: a randomized, multicenter, double-blind, placebo-controlled, parallel-group, dose-dependent clinical trial. GeroScience 45, 29–43 (2023). 36482258 10.1007/s11357-022-00705-1 PMC9735188

[33] Lee E. Exploring the effects of Dasatinib, Quercetin, and Fisetin on DNA methylation clocks: a longitudinal study on senolytic interventions. Aging 16, 3088–3106 (2024). 38393697 10.18632/aging.205581 PMC10929829

[34] Armenise C. Transcriptome profiling from adipose tissue during a low-calorie diet reveals predictors of weight and glycemic outcomes in obese, nondiabetic subjects. The American Journal of Clinical Nutrition 106, 736–746 (2017). 28793995 10.3945/ajcn.117.156216

[35] Benton M. C. An analysis of DNA methylation in human adipose tissue reveals differential modification of obesity genes before and after gastric bypass and weight loss. Genome Biology 16, 8 (2015). 25651499 10.1186/s13059-014-0569-x PMC4301800

[36] Castañer O. In vivo transcriptomic profile after a Mediterranean diet in high–cardiovascular risk patients: a randomized controlled trial123. The American Journal of Clinical Nutrition 98, 845–853 (2013). 23902780 10.3945/ajcn.113.060582

[37] McFarland A. J., Ray P. R., Bhai S., Levine B. D. & Price T. J. RNA sequencing on muscle biopsy from a 5-week bed rest study reveals the effect of exercise and potential interactions with dorsal root ganglion neurons. Physiological Reports 10, e15176 (2022). 35133080 10.14814/phy2.15176 PMC8823189

[38] Pheiffer C. Changes in subcutaneous adipose tissue microRNA expression in response to exercise training in African women with obesity. Sci Rep 12, 18408 (2022). 36319747 10.1038/s41598-022-23290-x PMC9626597

[39] Rönn T. A Six Months Exercise Intervention Influences the Genome-wide DNA Methylation Pattern in Human Adipose Tissue. PLOS Genetics 9, e1003572 (2013). 23825961 10.1371/journal.pgen.1003572 PMC3694844

[40] Maegawa S. Caloric restriction delays age-related methylation drift. Nat Commun 8, 1–11 (2017). 28912502 10.1038/s41467-017-00607-3 PMC5599616

[41] Mattison J. A. Impact of caloric restriction on health and survival in rhesus monkeys from the NIA study. Nature 489, 318–321 (2012). 22932268 10.1038/nature11432 PMC3832985

[42] Weindruch R., Kayo T., Lee C.-K. & Prolla T. A. Microarray profiling of gene expression in aging and its alteration by caloric restriction in mice. The Journal of nutrition 131, 918–S923S (2001). 10.1093/jn/131.3.918S 11238786

[43] Lu A. T. DNA methylation GrimAge strongly predicts lifespan and healthspan. aging 11, 303–327 (2019). 30669119 10.18632/aging.101684 PMC6366976

[44] Bareja A. Liver-derived plasminogen mediates muscle stem cell expansion during caloric restriction through the plasminogen receptor Plg-RKT. Cell Reports 43, 113881 (2024). 38442019 10.1016/j.celrep.2024.113881 PMC11075744

[45] Domcke S., Sinha R., Levine D. A., Sander C. & Schultz N. Evaluating cell lines as tumour models by comparison of genomic profiles. Nat Commun 4, 2126 (2013). 23839242 10.1038/ncomms3126 PMC3715866

[46] Oh J. H. & Cho J.-Y. Comparative oncology: overcoming human cancer through companion animal studies. Exp Mol Med 55, 725–734 (2023). 37009802 10.1038/s12276-023-00977-3 PMC10167357

[47] Redman L. M. Metabolic Slowing and Reduced Oxidative Damage with Sustained Caloric Restriction Support the Rate of Living and Oxidative Damage Theories of Aging. Cell Metabolism (2018) doi:10.1016/j.cmet.2018.02.019. PMC5886711 29576535

[48] Spadaro O. Caloric restriction in humans reveals immunometabolic regulators of health span. Science 375, 671–677 (2022). 35143297 10.1126/science.abg7292 PMC10061495

[49] Chen Q. OMICmAge: An integrative multi-omics approach to quantify biological age with electronic medical records. 2023.10.16.562114 Preprint at 10.1101/2023.10.16.562114 (2023).

[50] Lu A. T. DNA methylation GrimAge version 2. Aging 14, 9484–9549 (2022). 36516495 10.18632/aging.204434 PMC9792204

[51] Sehgal R. Systems Age: A Single Blood Methylation Test to Quantify Aging Heterogeneity across 11 Physiological Systems. http://biorxiv.org/lookup/doi/10.1101/2023.07.13.548904 (2023) doi:10.1101/2023.07.13.548904. PMC13222069 40954326

[52] Ying K. Causality-enriched epigenetic age uncouples damage and adaptation. Nat Aging 1–16 (2024) doi:10.1038/s43587-023-00557-0. 38243142 PMC11070280

[53] Fong S. The Principal Component-Based Clinical Aging Clock (PCAge) Identifies Signatures of Healthy Aging and Provides Normative Targets for Clinical Intervention. http://medrxiv.org/lookup/doi/10.1101/2023.07.14.23292604 (2023) doi:10.1101/2023.07.14.23292604.

[54] Kwon D. & Belsky D. W. A toolkit for quantification of biological age from blood chemistry and organ function test data: BioAge. GeroScience 43, 2795–2808 (2021). 34725754 10.1007/s11357-021-00480-5 PMC8602613

[55] Rohart F., Gautier B., Singh A. & Cao K.-A. L. mixOmics: An R package for ‘omics feature selection and multiple data integration. PLOS Computational Biology 13, e1005752 (2017). 29099853 10.1371/journal.pcbi.1005752 PMC5687754

[56] Wang B. Similarity network fusion for aggregating data types on a genomic scale. Nature Methods 11, 333–337 (2014). 24464287 10.1038/nmeth.2810

[57] Krassowski M., Das V., Sahu S. K. & Misra B. B. State of the Field in Multi-Omics Research: From Computational Needs to Data Mining and Sharing. Front. Genet. 11, (2020). 10.3389/fgene.2020.610798 PMC7758509 33362867

[58] Subramanian I., Verma S., Kumar S., Jere A. & Anamika K. Multi-omics Data Integration, Interpretation, and Its Application. Bioinform Biol Insights 14, 1177932219899051 (2020). 32076369 10.1177/1177932219899051 PMC7003173

[59] Bergström J. Percutaneous needle biopsy of skeletal muscle in physiological and clinical research. Scandinavian journal of clinical and laboratory investigation 35, 609–616 (1975). 1108172

[60] Sherry S. T. dbSNP: the NCBI database of genetic variation. Nucleic Acids Research 29, 308–311 (2001). 11125122 10.1093/nar/29.1.308 PMC29783

[61] Howie B., Marchini J. & Stephens M. Genotype Imputation with Thousands of Genomes. G3 Genes|Genomes|Genetics 1, 457–470 (2011). 22384356 10.1534/g3.111.001198 PMC3276165

[62] The 1000 Genomes Project Consortium A global reference for human genetic variation. Nature 526, 68–74 (2015). 26432245 10.1038/nature15393 PMC4750478

[63] Purcell S. PLINK: A Tool Set for Whole-Genome Association and Population-Based Linkage Analyses. The American Journal of Human Genetics 81, 559–575 (2007). 17701901 10.1086/519795 PMC1950838

[64] Davis S., Bilke S., Triche T. Jr & Bootwalla M. methylumi: Handle Illumina methylation data. (2024).

[65] Huber W. Orchestrating high-throughput genomic analysis with Bioconductor. Nature Methods 12, 115–121 (2015). 25633503 10.1038/nmeth.3252 PMC4509590

[66] Aryee M. J. Minfi: a flexible and comprehensive Bioconductor package for the analysis of Infinium DNA methylation microarrays. Bioinformatics 30, 1363–1369 (2014). 24478339 10.1093/bioinformatics/btu049 PMC4016708

[67] Salas L. A. An optimized library for reference-based deconvolution of whole-blood biospecimens assayed using the Illumina HumanMethylationEPIC BeadArray. Genome Biology 19, 64 (2018). 29843789 10.1186/s13059-018-1448-7 PMC5975716

[68] Zheng S. C. A Novel Cell-Type Deconvolution Algorithm Reveals Substantial Contamination by Immune Cells in Saliva, Buccal and Cervix. Epigenomics 10, 925–940 (2018). 29693419 10.2217/epi-2018-0037

[69] Higgins-Chen A. T. A computational solution for bolstering reliability of epigenetic clocks: implications for clinical trials and longitudinal tracking. Nat Aging 2, 644–661 (2022). 36277076 10.1038/s43587-022-00248-2 PMC9586209

[70] Daley T. & Smith A. D. Predicting the molecular complexity of sequencing libraries. Nature methods 10, 325–327 (2013). 23435259 10.1038/nmeth.2375 PMC3612374

[71] Wang L., Wang S. & Li W. RSeQC: quality control of RNA-seq experiments. Bioinformatics 28, 2184–2185 (2012). 22743226 10.1093/bioinformatics/bts356

[72] Bushnell B., Rood J. & Singer E. BBMerge–accurate paired shotgun read merging via overlap. PloS one 12, e0185056 (2017). 29073143 10.1371/journal.pone.0185056 PMC5657622

[73] Dobin A. STAR: ultrafast universal RNA-seq aligner. Bioinformatics 29, 15–21 (2013). 23104886 10.1093/bioinformatics/bts635 PMC3530905

[74] Liao Y., Smyth G. K. & Shi W. featureCounts: an efficient general purpose program for assigning sequence reads to genomic features. Bioinformatics 30, 923–930 (2014). 24227677 10.1093/bioinformatics/btt656

[75] Liao Y., Smyth G. K. & Shi W. The Subread aligner: fast, accurate and scalable read mapping by seed-and-vote. Nucleic Acids Research 41, e108–e108 (2013). 23558742 10.1093/nar/gkt214 PMC3664803

[76] Torgerson W. S. Multidimensional scaling: I. Theory and method. Psychometrika 17, 401–419 (1952).

[77] Everitt B. S., Landau S., Leese M. & Stahl D. Cluster Analysis. (John Wiley & Sons, 2011).

[78] Ritchie M. E. limma powers differential expression analyses for RNA-sequencing and microarray studies. Nucl. Acids Res. 43, e47–e47 (2015). 25605792 10.1093/nar/gkv007 PMC4402510

[79] Warnes M. G. R. Package ‘gplots’. Various R programming tools for plotting data (2016).

[80] Tibshirani R., Walther G. & Hastie T. Estimating the number of clusters in a data set via the gap statistic. Journal of the Royal Statistical Society: Series B (Statistical Methodology) 63, 411–423 (2001).

[81] Charrad M., Ghazzali N., Boiteau V. & Niknafs A. NbClust: an R package for determining the relevant number of clusters in a data set. Journal of statistical software 61, 1–36 (2014).

[82] Robinson M. D. & Oshlack A. A scaling normalization method for differential expression analysis of RNA-seq data. Genome Biol 11, 1–9 (2010). 10.1186/gb-2010-11-3-r25 PMC2864565 20196867

[83] Law C. W., Chen Y., Shi W. & Smyth G. K. voom: precision weights unlock linear model analysis tools for RNA-seq read counts. Genome Biol 15, 1–17 (2014). 10.1186/gb-2014-15-2-r29 PMC4053721 24485249

[84] Liu R. Why weight? Modelling sample and observational level variability improves power in RNA-seq analyses. Nucleic acids research 43, e97–e97 (2015). 25925576 10.1093/nar/gkv412 PMC4551905

[85] Law C. W. A guide to creating design matrices for gene expression experiments. F1000Res 9, 1444 (2020). 33604029 10.12688/f1000research.27893.1 PMC7873980

[86] Smyth G. K., Michaud J. & Scott H. S. Use of within-array replicate spots for assessing differential expression in microarray experiments. Bioinformatics 21, 2067–2075 (2005). 15657102 10.1093/bioinformatics/bti270

[87] Benjamini Y. & Hochberg Y. Controlling the false discovery rate: a practical and powerful approach to multiple testing. Journal of the royal statistical society. Series B (Methodological) 289–300 (1995).

[88] Korotkevich G. Fast gene set enrichment analysis. 060012 Preprint at 10.1101/060012 (2021).

[89] Luo W., Friedman M. S., Shedden K., Hankenson K. D. & Woolf P. J. GAGE: generally applicable gene set enrichment for pathway analysis. BMC Bioinformatics 10, 161 (2009). 19473525 10.1186/1471-2105-10-161 PMC2696452

